# Effect of collagen matrix on postoperative palatal fistula in cleft palate repair

**DOI:** 10.1038/s41598-020-72046-y

**Published:** 2020-09-17

**Authors:** Jeong Hyun Ha, Yeonwoo Jeong, Youn Taek Koo, Sungmi Jeon, Jeehyeok Chung, Sukwha Kim

**Affiliations:** 1 Department of Plastic and Reconstructive Surgery, Seoul Metropolitan Government-Seoul National University Boramae Medical Center, Seoul, Korea; 2 Slow Plastic Surgery, Jeju, Korea; 3 Pop Plastic Surgery, Seoul, Korea; 4 Department of Plastic and Reconstructive Surgery, Seoul National University Hospital, Seoul, Korea; 5 Division of Pediatric Plastic Surgery, Seoul National University Children’s Hospital, Seoul, Korea; 6 Department of Plastic and Reconstructive Surgery, Seoul National University College of Medicine, Seoul, Korea; 7 Medical Big Data Research Center, Seoul, Korea

**Keywords:** Oral diseases, Outcomes research, Paediatric research

## Abstract

Palatal fistula is a challenging complication following cleft palate repair. We investigated the usefulness of collagen matrix in the prevention of postoperative fistula. We performed a retrospective cohort study of patients with cleft palate who underwent primary palatoplasty (Furlow’s double opposing z-plasty) in Seoul National University Children’s Hospital. Collagen Graft and Collagen Membrane (Genoss, Suwon, Republic of Korea) were selectively used in patients who failed complete two-layer closure. The effect of collagen matrix on fistula formation was evaluated according to palatal ratio (cleft width to total palatal width) and cleft width. A total of 244 patients (male, 92 and female, 152; median age, 18 months) were analyzed. The average cleft width was 7.0 mm, and the average palatal ratio was 0.21. The overall fistula rate was 3.6% (9/244). Palatal ratio (p = 0.014) and cleft width (p = 0.004) were independent factors impacting the incidence of postoperative fistula. Receiver operating characteristic curve analysis showed that the cutoff values in terms of screening for developing postoperative fistula were a palatal ratio of 0.285 and a cleft width of 9.25 mm. Among nonsyndromic patients with values above those cutoffs, the rates of fistula development were 0/5, 1/6 (16.7%), and 4/22 (18.2%) for those who received Collagen Graft, Collagen Membrane, and no collagen, respectively. Collagen matrix may serve as an effective tool for the prevention of palatal fistula when complete two-layer closure fails, especially in wide palatal clefts. The benefit was most evident in Collagen Graft with thick and porous structure.

## Introduction

Techniques for cleft palate repair have evolved through years, but postoperative palatal fistula remains a challenging complication. Postoperative palatal fistula results from unsuccessful wound healing due to excessive tension, infection, or hematoma. Wide palatal clefts are prone to excessive tension during closure, making them especially vulnerable to postoperative fistula development^1,2^. The rate of postoperative fistula formation might differ according to the surgical method, but it generally ranges from 2.4 to 35%^2^. Furlow’s double opposing z-plasty (DOZ palatoplasty) has the potential to provide superior speech outcomes compared with other methods of cleft palate repair^3^, but it is also prone to the development of tension during wound closure, which can lead to postoperative fistula^4^. It is also prone to the formation of a large dead space between the oral and nasal linings after cleft repair, which is likely to be filled with hematoma, resulting in fistula.

Surgical repair of postoperative fistula is difficult because of inevitable scarring, altered vascularity, and persistent tension after primary palatoplasty^1^. Even after repair, the recurrence rate of fistula is high, ranging from 9 to 65%^5^. Palatal fistula can cause nasal regurgitation or inflammatory irritation and can also have adverse effects on speech, including nasal emission and backing of alveolar consonants. Some surgeons report that fistulas have detrimental effects on soft palate function, resulting in velopharyngeal dysfunction^4,6^. Therefore, it is important to take measures to prevent the formation of postoperative palatal fistula.

There are numerous techniques to prevent and treat postoperative palatal fistula using local or distant tissues^5,7–12^. Attempts have also been made to develop synthetic biomaterials that can decrease the incidence of postoperative fistula^13^. There are several recent reports of favorable outcomes after the use of acellular dermal matrix (ADM)^4,5,14,15^. Collagen matrix has also been used for palatal fistula repair, resulting in successful outcomes and decreased recurrence rates^6,16^.

Here, we present our experiences using two types of resorbable collagen matrix [Collagen Membrane, Collagen Graft (Genoss, Suwon, Republic of Korea)] as an interposition graft in cases where complete two-layer closure fails in primary DOZ palatoplasty. Our objective was to delineate the usefulness of collagen matrix to prevent or decrease the development of fistula after cleft palate repair.

## Patients and methods

We conducted a retrospective cohort study of patients with cleft palate who underwent primary DOZ palatoplasty at Seoul National University Children’s Hospital from July 2015 to March 2018. Patients who had undergone previous palatal surgery (e.g., teratoma or mass excision) were excluded. Patients with comorbidities were also excluded. After receiving approval from the Seoul National University Hospital Institutional Review Board (IRB No. H-1805-016-944), we reviewed the patients’ demographic data, medical information, and photographs. Postoperative palatal fistula was categorized according to the Pittsburgh Fistula Classification System^17^. Owing to our study design, we got waiver for informed consent by Seoul National University Hospital Institutional Review Board following the institution’s policy. The recommendations of the Declaration of Helsinki for biomedical research involving human subjects were followed. All methods were carried out in accordance with relevant guidelines and regulations.

### Operative technique

**Figure 1:**
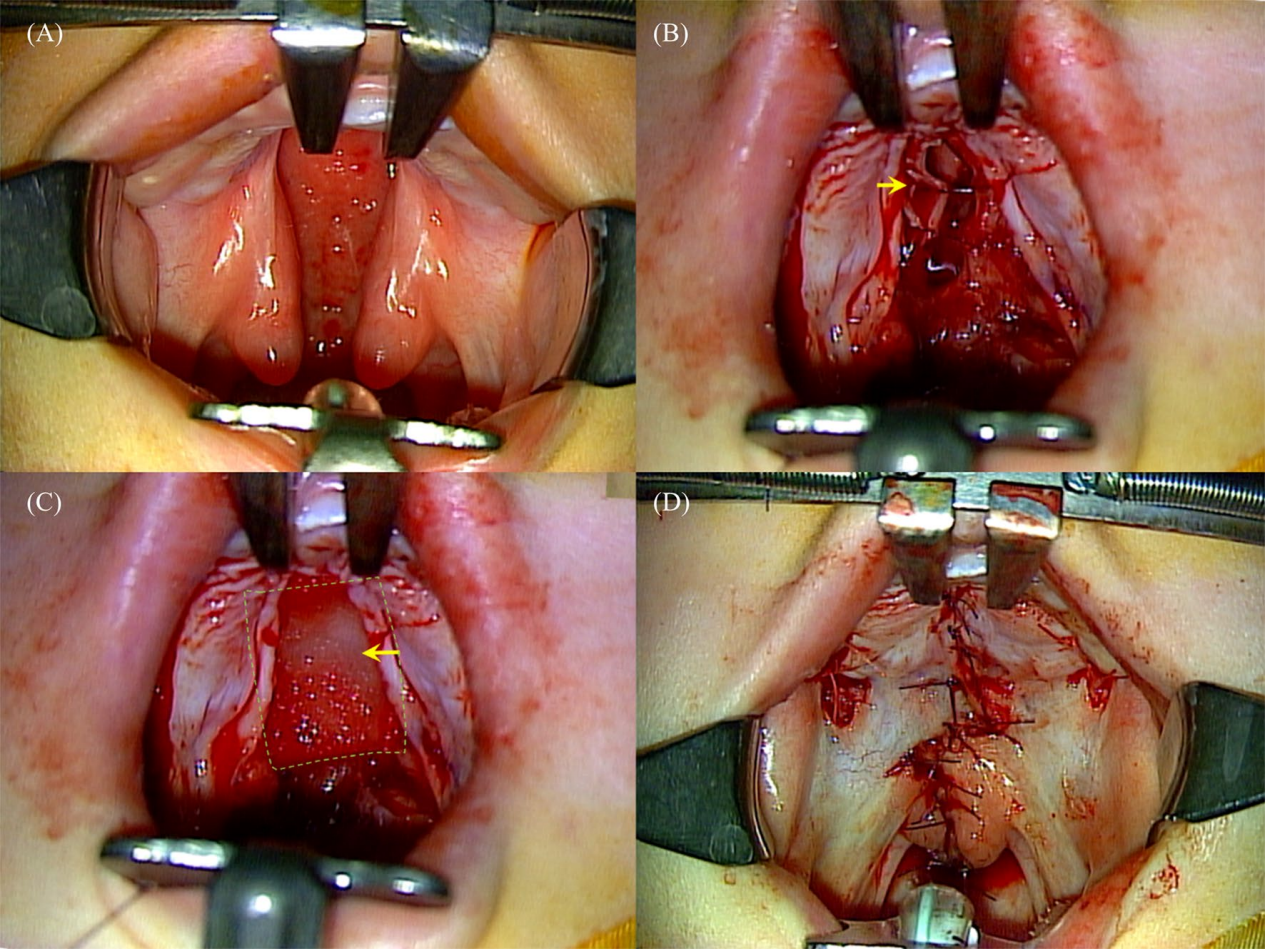
(**A**) Eleven-month-old female patient with Veau type II cleft palate. The palatal ratio was 0.32. The cleft width was 12 mm. (**B**) Furlow’s double opposing z-palatoplasty was performed. Oral and nasal flaps were created, and bilateral relaxing incisions were made. Complete nasal lining closure failed because of high tension. (**C**) Collagen matrix (arrow) was used over the nasal lining. (**D**) Postoperative photo of Furlow’s double opposing Z-palatoplasty.

All patients underwent DOZ palatoplasty. In cases with wide palatal clefts, lateral relaxing incisions were made in the gingival hard palate on one or both sides to facilitate wound closure. Vomer flaps were also considered when wound approximation was insufficient. During the procedure, we used Collagen Membrane or Collagen Graft resorbable collagen matrix as an interposition graft when there was inappropriate approximation of the nasal lining causing failure of complete two-layer closure (Fig. 1). Initially, we used Collagen Membrane (0.3 mm thickness, single layer) as the collagen matrix, but we determined that the more supportive material would be beneficial; therefore, we switched to Collagen Graft (3 mm thickness, double layer).

All patients consumed a soft diet for 1 month postoperatively. Arm splint was applied for 1 month for surgical wound protection. Postoperative follow-up was performed at 1 week, 1 month, 3 months, and 6 months after surgery. In cases of palatal fistula formation, additional follow-up was performed every 3 months. If there was no palatal fistula formation, routine follow-up for speech evaluation was performed when the patients reached 3 years of age. (Supplementary Fig. S1 shows follow-up cases of postoperative dehiscence leading to permanent fistula or spontaneously healed).

### Evaluation

**Figure 2:**
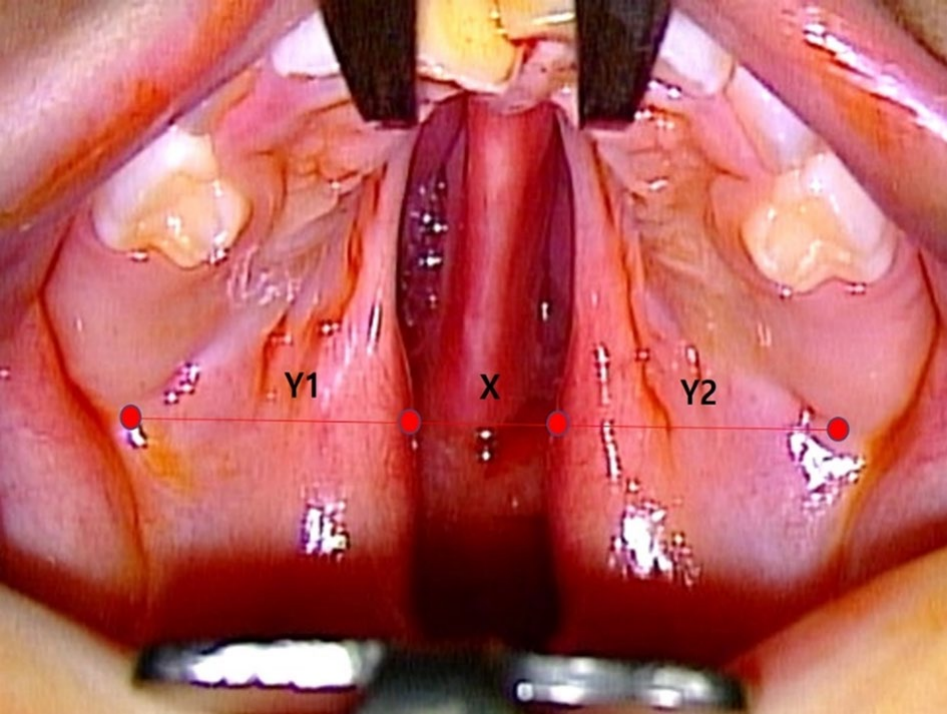
Palatal measurements. Palatal ratio is defined as the ratio of cleft width to total palatal width [X/X + Y1 + Y2]. Each parameter was measured at the level of maxillary tuberosity.

The cleft width (X) and palatal shelf widths (Y1, Y2) were measured preoperatively with the patients under general anesthesia. We obtained each parameter from the patients’ medical records and calculated the palatal ratio, or the ratio of the cleft width to the total palatal width, as X/(X + Y1 + Y2) (Fig. 2). We divided the patients into three groups. The first group did not receive any collagen matrix. The other two groups received either Collagen Membrane or Collagen Graft.

### Statistical analysis

We performed statistical analyses using logistic regression model to analyze the effects of continuous numeric variables on clinical outcomes. We assessed factors affecting the risk of postoperative fistula only among the nonsyndromic patients that did not receive collagen matrix in order to exclude any potential influence of the collagen matrix or the syndromic condition^1^. We used receiver operating characteristic curve (ROC) analysis to estimate the cutoff values of palatal ratio and cleft width that indicated an increased risk of postoperative fistula. We then identified subgroups with high risk of postoperative fistula development using the cutoff values. All statistical tests were two-sided, and significance was defined by p < 0.05. All analyses were performed using the Statistical Package for the Social Sciences for Windows Version 21.0 (IBM, Chicago, IL, USA).

## Results

### Patient characteristics

A total of 244 patients with cleft palate (92 males, 152 females) underwent palatoplasty during the study period. Twenty-eight of the patients had accompanying conditions. The proportions of patients by Veau classification were 1.6% submucous cleft palate, 21.7% Veau type I, 61.5% Veau type II, 12.3% Veau type III, and 2.9% Veau type IV. All operations were performed by one experienced plastic surgeon (Sukwha Kim). The mean age at the time of surgery was 17.5 months (range, 9–105 months). The average follow-up duration was 26.1 months (range, 9–42 months). The average (± standard deviation) cleft width was 7.0 (± 3.5) mm, and the average (± standard deviation) palatal ratio was 0.21 (± 0.10). Collagen matrix was used in 33 patients (14.7%) as follows: 1 Veau type I, 30 Veau type II, and 2 Veau type III. (Table 1) Postoperative palatal fistula was observed in 9 patients overall (3.6%) (Table 2). There were no major complications other than postoperative palatal fistula.

**Table 1:** Patient demographics.

Characteristics	Non-collagen group	Collagen membrane group	Collagen graft group	p-value
Age, months	17.23 (10–105)	13.78 (9–22)	15.65 (11–26)	0.091*
**Sex**				
Male	71	10	2	
Female	117	10	7	
**Veau classification**				
Submucous cleft palate	4	0	0	
I	47	0	0	
II	107	18	9	
III	25	2	0	
IV	4	0	0	
Cleft width, mm	6.61 (SD 3.06)	9.45 (SD 3.07)	10.44 (SD 2.65)	< 0.001*
Palatal ratio	0.20 (SD 0.09)	0.26 (SD 0.07)	0.31 (SD 0.12)	< 0.001*
Fistula (n)	7	2	0	

*Kruskal–Wallis test.

Palatal ratio, cleft width–to–total palatal width ratio; Palatal index, cleft width-to-maxillary width ratio.

**Table 2:** Clinical series of patients who developed postoperative fistula.

Patient	Collagen matrix usage	Location of Fistula	Size of Fistula (mm)
1	None	Hard palate posterior 1/3	5 × 1
2	None	Hard-soft palate junction	1.5 × 0.8
3	None	Hard palate posterior 1/3	3 × 1.5
4	Collagen Membrane	Hard palate posterior 1/3	4 × 1
5	None	Hard palate posterior 1/3	4 × 3
6	None	Lt. premaxilla	3 × 0.8
7	Collagen Membrane	Hard-soft palate junction	1 × 1
8	None	Hard-soft palate junction	2 × 0.3
9	None	Hard palate posterior 1/2	10 × 1

### Factors affecting Fistula formation

We first evaluated the association between cleft severity and postoperative fistula formation among the nonsyndromic patients that did not receive collagen matrix. The fistula rate was significantly higher among patients with Veau type III or type IV clefts than among those with Veau type I or type II clefts (p = 0.029), suggesting that the fistula rate increased with the severity of the cleft.

We next analyzed the association between postoperative fistula formation and the cleft width and palatal parameters among the nonsyndromic patients that did not receive collagen matrix. The palatal ratio and cleft width were both independent risk factors for postoperative fistula occurrence (p = 0.014 and p = 0.004, respectively).

**Figure 3:**
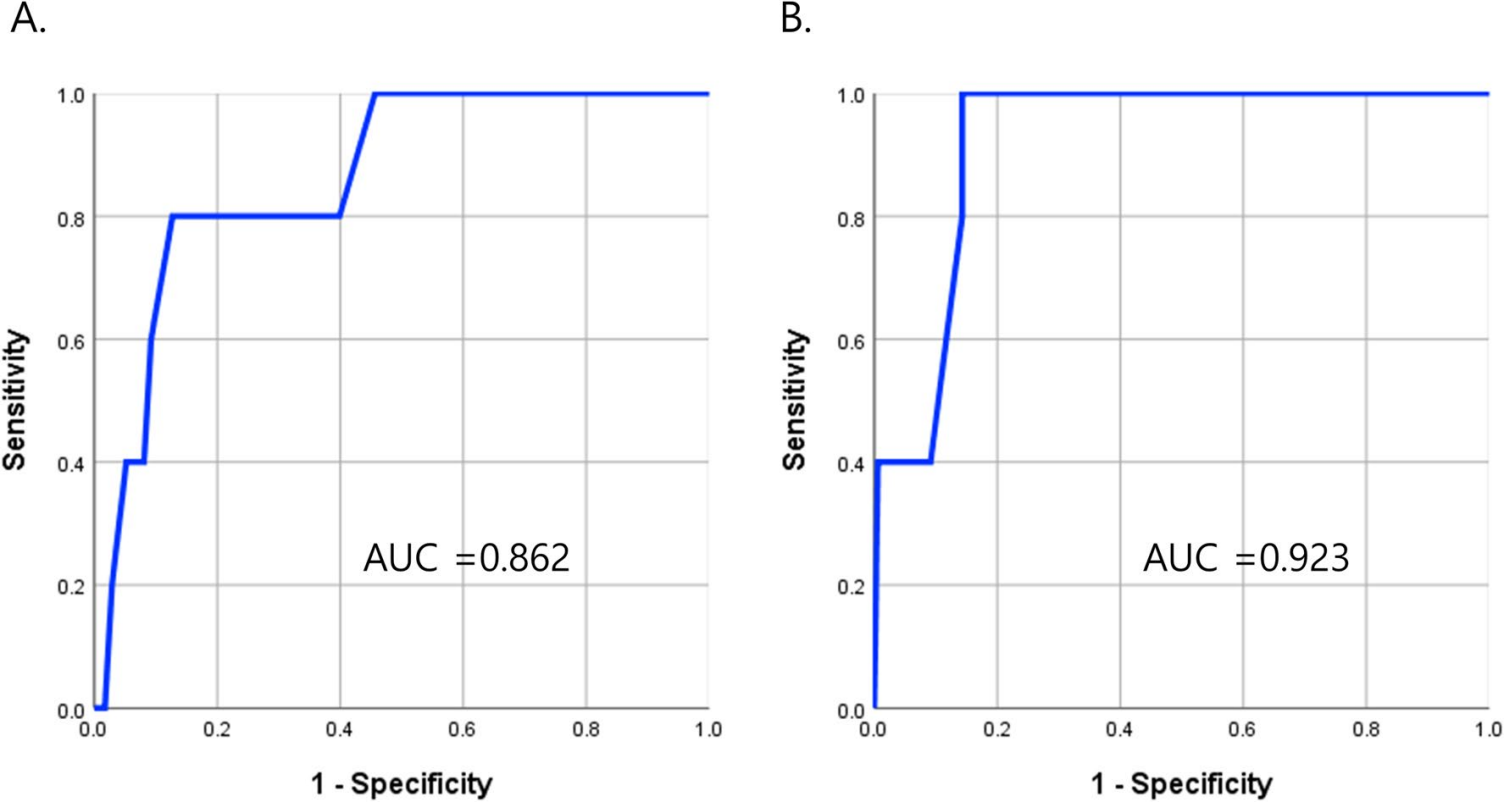
Receiver operating characteristic curve (ROC) analysis to estimate the cutoff values of (**A**) palatal ratio (0.285) and (**B**) cleft width (9.25 mm) indicating increased risk of postoperative fistula.

We determined the cut-off values of cleft width and palatal ratio in terms of screening for developing postoperative fistula. The cut-off values for screening were 0.285 of palatal ratio (AUC, Area Under Curve = 0.862) and 9.25 mm of cleft width (AUC = 0.923) from ROC analysis. (Fig. 3).

### Collagen matrix

Twenty-two patients received Collagen Membrane. The patients that received Collagen Membrane had a mean age of 16 (± 4.4) months, an average cleft width of 9.4 (± 3.06) mm, and an average palatal ratio of 0.26 (± 0.75). Two patients developed postoperative fistula after receiving Collagen Membrane. Eleven patients received Collagen Graft. The patients that received Collagen Graft had a mean age of 15.6 (± 6.77) months, an average cleft width of 10.09 (± 2.51) mm, and an average palatal ratio of 0.30 (± 0.11). One syndromic patient developed postoperative fistula after receiving Collagen Graft. None of the patients that received collagen matrix experienced complications involving infection or local inflammation. Because of the relatively short follow-up, speech evaluation could not be performed in all patients. 26 nonsyndromic patients received collagen matrix of which 24 patients (14, Collagen Membrane; 7, Collagen Graft) underwent speech evaluation. Among these patients, 3 patients showed hypernasality and 3 patients showed compensatory articulation.

### Role of collagen matrix in high-risk subgroups

**Figure 4:**
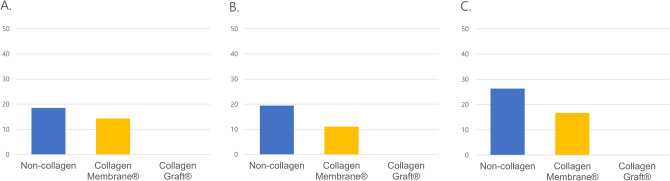
Postoperative fistula rate (%) in subgroups with (**A**) high palatal ratio (> 0.285), (**B**) large cleft width (> 9.25 mm), and (**C**) high palatal ratio (> 0.285) and large cleft width (> 9.25 mm).

Next, we examined the outcomes of Collagen Membrane and Collagen Graft use in nonsyndromic patients that had a high risk of postoperative fistula because of a high palatal ratio, a large cleft width, or both. Among the 39 patients that had a high palatal ratio (> 0.285), the rate of postoperative fistula formation was 1/13 (7.7%) among those that received collagen matrix (0/6, Collagen Graft; 1/7, Collagen Membrane), and 4/26 (15.4%) among those that received no collagen matrix. Among the 45 patients that had a cleft width > 9.25 mm, the rate of postoperative fistula formation was 1/15 (6.7%) among that received collagen matrix (0/6, Collagen Graft; 1/9, Collagen Membrane), and 5/30 (16.7%) among those that received no collagen matrix. Among the 29 patients who had both a high palatal ratio and a large cleft width, the rate of postoperative fistula formation was 1/11 (9.1%) among those that received collagen matrix (0/5, Collagen Graft; 1/6, Collagen Membrane), and 4/18 (22.2%) among those that received no collagen matrix. None of the patients in any of the three high-risk subgroups developed postoperative fistula after using Collagen Graft (Fig. 4).

## Discussion

The ideal cleft palate repair is a tension-free, water-tight closure. It is important to achieve two-layer closure without tension to reduce the incidence of postoperative fistula. Postoperative fistula is more likely to occur in patients with a wide palatal cleft, arising from excessive tension or a large dead space^1,18^. Failure of complete two-layer closure is not rare after DOZ palatoplasty of wide palatal clefts, increasing the likelihood of postoperative fistula formation^19^. Nevertheless, DOZ palatoplasty provides superior speech outcomes compared with other techniques^3^. DOZ palatoplasty simultaneously lengthens the soft palate and mobilizes the levator muscle posteriorly, forming the muscle sling. Moreover, DOZ palatoplasty only requires muscle dissection in one surface, leaving the other surface attached with the mucosa, resulting in less fibrosis and scarring^20^. For those reasons, we perform DOZ palatoplasty as the primary technique for cleft palate repair.

Besides the absolute value of the cleft width, a higher ratio of cleft width to the total palatal width is associated with a higher incidence of postoperative fistula^21^. Large cleft width and high palatal ratio both affected postoperative fistula development in our patients after DOZ palatoplasty. We found that a cleft width of 9.25 mm and a palatal ratio of 0.285 were the cutoff values for screening patients who might develop a postoperative fistula. To exclude any effects from the collagen matrix, we performed the ROC analysis using only the patients that had not receive collagen matrix. However, there is a possibility of underestimation in the value of cleft width and palatal ratio. The value of the widest part of the cleft is a powerful predictor for postoperative fistula formation. Unfortunately, our data for cleft width was measured at the level of maxillary tuberosity, which may not always correspond to the widest part (Supplementary Fig. S2 shows a comparison between the cleft width at the level of maxillary tuberosity (yellow) and at the widest level (green)). On the other hand, the cut-off value we set may not truly be the threshold value for postoperative fistula. Considering the incidence, the cut-off values for a high risk of postoperative fistula were 12.5 mm of cleft width and 0.345 of palatal ratio, adding more weight on specificity.

Numerous efforts have been made to decrease the incidence of postoperative fistula. Relaxing incisions, extensive dissection, and hamulus fracture may be utilized to acquire sufficient soft tissue for closure. In complicated cases, tongue flaps or buccal flaps may also be tried. Those techniques require two stages, however, and are usually used in secondary palatoplasty after postoperative fistula. Various synthetic materials have also been applied. Recently, the use of ADM in primary or secondary palatoplasty has shown favorable results in terms of reducing the incidence of postoperative palatal fistula^1,4,5^. Although no major complications after ADM use have been reported, there are still potential disadvantages including velum scarring and infection, which might also lead to adverse speech outcomes. The use of resorbable collagen materials is another approach to reduce the risk of postoperative fistula. Atherton et al.^6^ reported that the use of collagen matrix in palatal fistula repair resulted in improved outcomes. Sader et al.^16^ reported no recurrent fistulas after palatal fistula repair using collagen matrix in 14 patients. Hudson et al.^22^ used collagen matrix (type 1 collagen, 0.5 mm thickness) in primary palatoplasty in six patients and reported no fistula occurrence.

ADM or collagen materials were only used in secondary palatoplasty or in a limited number of cases of primary palatoplasty in previous studies. We investigated the usefulness of collagen matrix to prevent postoperative fistula in primary palatoplasty when complete two-layer closure was not possible. We used the collagen matrix (Collagen Membrane or Collagen Graft) as an interposition graft to serve as a mechanical barrier, volumetric spacer, and regenerative scaffold in wide palatal clefts^16^. Collagen Membrane and Collagen Graft are composed of type I collagen derived from bovine tendon. Bovine collagen is weakly antigenic because of high sequence homology between bovine and human collagen. Its safety has been validated in the orthodontic field for use after tooth extraction or as a barrier membrane^23,24^. It induces only a mild inflammatory reaction during degradation and has good biodegradable and biocompatible properties. The space occupied by the collagen material is replaced by host soft tissue within 4 to 12 weeks^23,25^. Excessive tension bears a risk of wound dehiscence. When complete two-layer closure fails, wound disruption leads directly to palatal fistula. In that fragile area, the collagen matrix functions as a mechanical barrier, preventing reepithelialization of the fistula tract and providing a scaffold for tissue regeneration^16^. At first, we used Collagen Membrane (0.3 mm thickness). However, that the thickness of the Collagen Membrane was not enough to fill the dead space between the oral and nasal layers. Furthermore, the dense structure of the Collagen Membrane did not easily induce host tissue ingrowth, resulting in a limited effect on fistula prevention. Therefore, we switched the graft material to Collagen Graft (3 mm thickness), which has a porous structure and provides better tissue regeneration and appropriate filling of the dead space.

When we analyzed the fistula rate in subgroups (palatal ratio > 0.285, cleft width > 9.25 mm, or both) there was no fistula formation among the patients that received Collagen Graft, whereas Collagen Membrane was not as effective in preventing fistula. Those results were not statistically significant, however, possibly because of the small sample size. Despite the lack of statistical difference, the total absence of postoperative fistula after Collagen Graft use suggests that Collagen Graft may decrease the risk of postoperative fistula. This should be verified in a larger sample of patients. We conjecture that Collagen Graft can be useful in patients who are susceptible to fistula development, not only when a complete two-layer closure is impossible, but also in clefts with a high palatal ratio or a large cleft width. Although collagen matrix was used only after failure of complete two-layer closure in our study, high palatal ratio or large cleft width might serve as indicators for its usage in primary palatoplasty. A larger study will be needed to reach a definitive conclusion.

Our study has several limitations. First, it is a retrospective study with a limited sample size. Some patients lacked palatal measurements, and detailed postoperative assessments were limited. Second, we could not identify the effect of collagen interposition grafting on maxillary growth, because the follow-up period was not long enough. We assume that collagen matrix also has a role in filling dead space. Long-term follow-up studies are needed to assess such outcome after the use of collagen matrix in cleft palate surgery. Moreover, our study did not include patients with the same indications who did not receive collagen matrix. A large-sized prospective randomized study is needed to draw firm conclusions about recommendations for the routine use of collagen matrix in high-risk patients.

## Conclusion

High palatal ratio and cleft width seem to be associated with higher fistula rates. Collagen matrix may help in the prevention of palatal fistula when a complete two-layer closure is impossible. Collagen Graft, with its thick and porous structure, appears to be more efficient in fistula prevention than the thinner Collagen Membrane.

## Supplementary information

Supplementary Figure 1.

Supplementary Figure 2.

## References

[1] Helling ER (2006). Low fistula rate in palatal clefts closed with the Furlow technique using decellularized dermis. Plast. Reconstr. Surg..

[2] Tse RW, Siebold B (2018). Cleft palate repair: description of an approach, its evolution, and analysis of postoperative Fistulas. Plast. Reconstr. Surg..

[3] Gunther E, Wisser JR, Cohen MA, Brown AS (1998). Palatoplasty: Furlow's double reversing Z-plasty versus intravelar veloplasty. Cleft Palate Craniofac. J..

[4] Losee JE (2008). A successful algorithm for limiting postoperative fistulae following palatal procedures in the patient with orofacial clefting. Plast. Reconstr. Surg..

[5] Kirschner RE (2006). Repair of oronasal fistulae with acellular dermal matrices. Plast. Reconstr. Surg..

[6] Atherton DD, Boorman JG (2016). Use of a purified collagen membrane to aid closure of palatal fistulae. J. Plast. Reconstr. Aesthet. Surg..

[7] Schultz RC (1986). Management and timing of cleft palate fistula repair. Plast. Reconstr. Surg..

[8] Assuncao AG (1993). The design of tongue flaps for the closure of palatal fistulas. Plast. Reconstr. Surg..

[9] Berkman MD (1978). Early non-surgical closure of postoperative palatal fistulae. Plast. Reconstr. Surg..

[10] Chen HC, Ganos DL, Coessens BC, Kyutoku S, Noordhoff MS (1992). Free forearm flap for closure of difficult oronasal fistulas in cleft palate patients. Plast. Reconstr. Surg..

[11] Denny AD, Amm CA (2005). Surgical technique for the correction of postpalatoplasty fistulae of the hard palate. Plast. Reconstr. Surg..

[12] Krimmel M, Hoffmann J, Reinert S (2005). Cleft palate fistula closure with a mucosal prelaminated lateral upper arm flap. Plast. Reconstr. Surg..

[13] Sharif, F.*, et al.* Developing a synthetic composite membrane for cleft palate repair. *J Tissue Eng Regen Med* (2019). 10.1002/term.2867 30977264

[14] Aldekhayel SA, Sinno H, Gilardino MS (2012). Acellular dermal matrix in cleft palate repair: an evidence-based review. Plast. Reconstr. Surg..

[15] Steele MH, Seagle MB (2006). Palatal fistula repair using acellular dermal matrix: the University of Florida experience. Ann. Plast. Surg..

[16] Sader R, Seitz O, Kuttenberger J (2010). Resorbable collagen membrane in surgical repair of fistula following palatoplasty in nonsyndromic cleft palate. Int. J. Oral. Maxillofac. Surg..

[17] Smith DM (2007). The Pittsburgh Fistula Classification System: a standardized scheme for the description of palatal fistulas. Cleft. Palate Craniofac. J..

[18] Parwaz MA (2009). Width of cleft palate and postoperative palatal fistula–do they correlate?. J. Plast. Reconstr. Aesthet. Surg..

[19] Losken HW (2011). Achieving low cleft palate fistula rates: surgical results and techniques. Cleft Palate Craniofac. J..

[20] Randall P, LaRossa D, Solomon M, Cohen M (1986). Experience with the Furlow double-reversing Z-plasty for cleft palate repair. Plast. Reconstr. Surg..

[21] Rossell-Perry P, Caceres Nano E, Gavino-Gutierrez AM (2014). Association between palatal index and cleft palate repair outcomes in patients with complete unilateral cleft lip and palate. JAMA Facial Plast. Surg..

[22] Hudson JW, Pickett DO (2015). A 5-year retrospective review of primary palatoplasty cases utilizing an acellular collagen interpositional graft. J. Oral. Maxillofac. Surg..

[23] Song YW (2019). Soft tissue substitutes to increase gingival thickness: histologic and volumetric analyses in dogs. J. Clin. Periodontol..

[24] Cha JK, Sun YK, Kim MJ, Sanz M, Jung UW (2018). Anti-resorptive effect of pamidronate on extraction socket wall in dogs. Clin. Oral. Implants Res..

[25] Jansen RG, Kuijpers-Jagtman AM, van Kuppevelt TH, Von den Hoff JW (2008). Collagen scaffolds implanted in the palatal mucosa. J. Craniofac. Surg..

