# Supplementary figures and images for: Effect of collagen matrix on postoperative palatal fistula in cleft palate repair
Source: Sci Rep. 2020 Sep 17;10:15236. doi: 10.1038/s41598-020-72046-y (PMC7498452; doi:10.1038/s41598-020-72046-y)

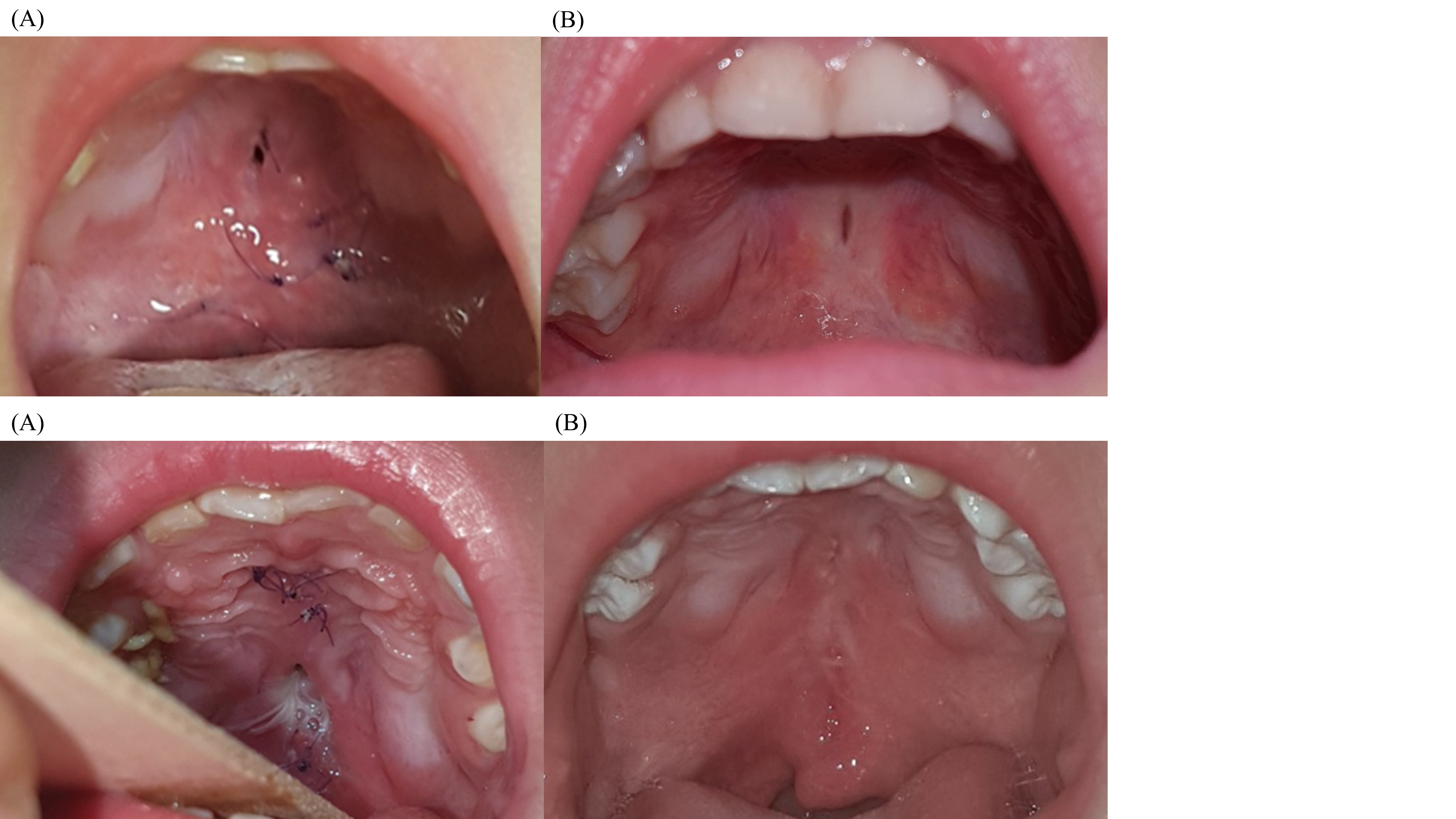

Supplement: Supplementary file 1 — Supplementary Figure 1. [file 41598_2020_72046_MOESM1_ESM.tif]

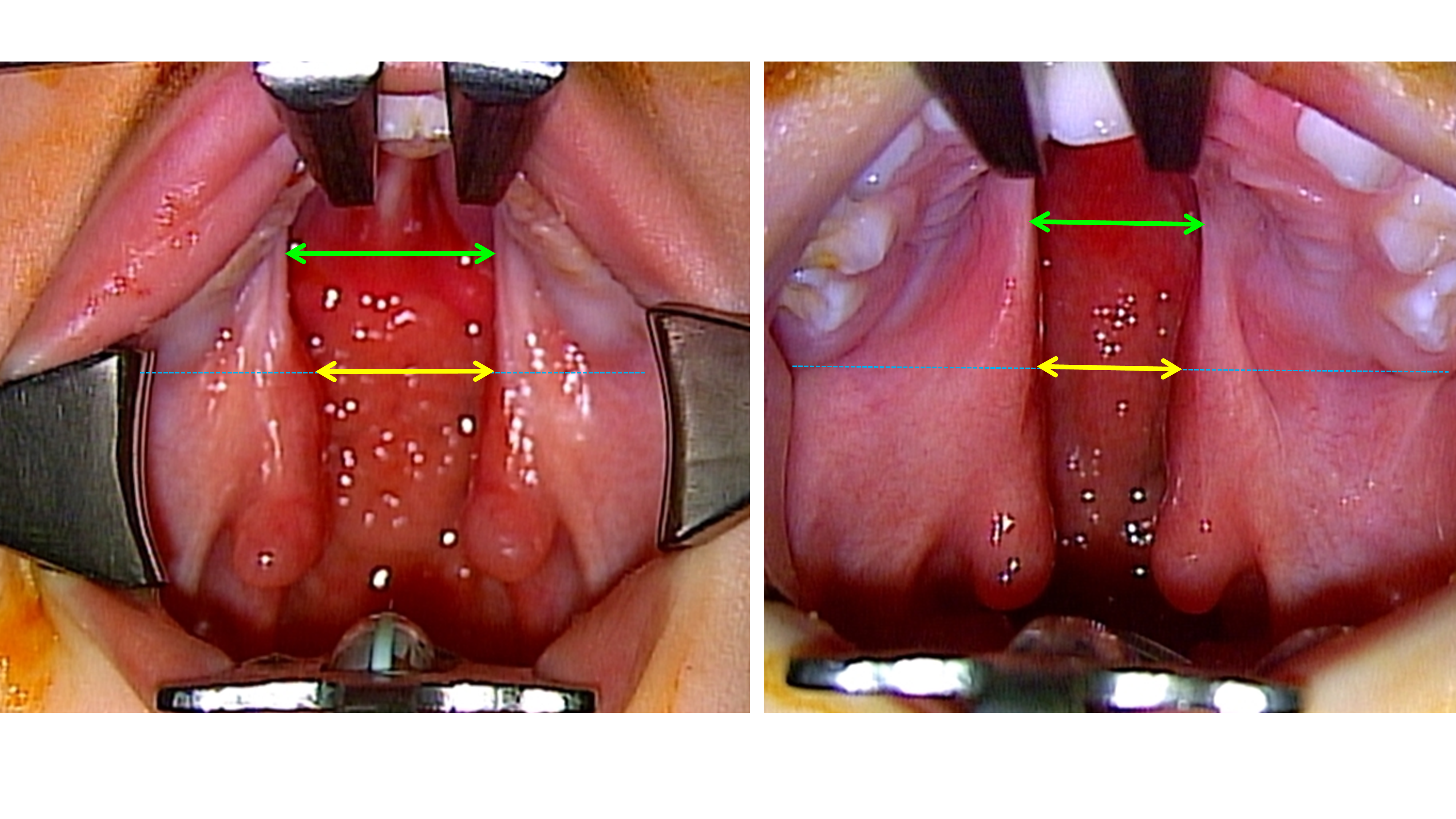

Supplement: Supplementary file 2 — Supplementary Figure 2. [file 41598_2020_72046_MOESM2_ESM.tif]
